# Temporal dynamics of muscle mitochondrial uncoupling-induced integrated stress response and ferroptosis defense

**DOI:** 10.3389/fendo.2023.1277866

**Published:** 2023-10-23

**Authors:** Carla Igual Gil, Alina Löser, Kristina Lossow, Maria Schwarz, Daniela Weber, Tilman Grune, Anna P. Kipp, Susanne Klaus, Mario Ost

**Affiliations:** ^1^ Department of Physiology of Energy Metabolism, German Institute of Human Nutrition Potsdam-Rehbrücke, Nuthetal, Germany; ^2^ Institute of Nutritional Science, University of Potsdam, Potsdam, Germany; ^3^ Department of Nutritional Physiology, Institute of Nutritional Sciences, Friedrich Schiller University Jena, Jena, Germany; ^4^ TraceAge-Deutsche Forschungsgemeinschaft (DFG) Research Unit on Interactions of Essential Trace Elements in Healthy and Diseased Elderly, Potsdam-Berlin-Jena-Wuppertal, Germany; ^5^ Department of Molecular Toxicology, German Institute of Human Nutrition Potsdam-Rehbrücke, Nuthetal, Germany; ^6^ Paul Flechsig Institute of Neuropathology, University Clinic Leipzig, Leipzig, Germany

**Keywords:** mitochondrial uncoupling, skeletal muscle, integrated stress response, FGF21, GDF15, ferroptosis, oxidative stress, circadian rhythm

## Abstract

Mitochondria play multifaceted roles in cellular function, and impairments across domains of mitochondrial biology are known to promote cellular integrated stress response (ISR) pathways as well as systemic metabolic adaptations. However, the temporal dynamics of specific mitochondrial ISR related to physiological variations in tissue-specific energy demands remains unknown. Here, we conducted a comprehensive 24-hour muscle and plasma profiling of male and female mice with ectopic mitochondrial respiratory uncoupling in skeletal muscle (m*Ucp1*-transgenic, TG). TG mice are characterized by increased muscle ISR, elevated oxidative stress defense, and increased secretion of FGF21 and GDF15 as ISR-induced myokines. We observed a temporal signature of both cell-autonomous and systemic ISR in the context of endocrine myokine signaling and cellular redox balance, but not of ferroptotic signature which was also increased in TG muscle. We show a progressive increase of muscle ISR on transcriptional level during the active phase (night time), with a subsequent peak in circulating FGF21 and GDF15 in the early resting phase. Moreover, we found highest levels of muscle oxidative defense (GPX and NQO1 activity) between the late active to early resting phase, which could aim to counteract excessive iron-dependent lipid peroxidation and ferroptosis in muscle of TG mice. These findings highlight the temporal dynamics of cell-autonomous and endocrine ISR signaling under skeletal muscle mitochondrial uncoupling, emphasizing the importance of considering such dissociation in translational strategies and sample collection for diagnostic biomarker analysis.

## 1 Introduction

Systemic regulation of energy balance is an intrinsically dynamic process which is governed by alternating phases of feeding, activity, and resting in a diurnal manner; and many of the involved biochemical and molecular processes are controlled by the circadian clock as a biological time-keeping system. Mitochondria, key multifunctional and dynamic organelles in health and disease (1), are integrative hubs of cellular energy and substrate metabolism and as such, their activity oscillates in synchrony with the diurnal fluctuations of cellular energy demands (2). Indeed, various aspects of mitochondrial biology are under the control of the circadian clock system (3, 4). Impairments in the multifaceted functions of mitochondria are related to a number of metabolic disorders and other diseases. In turn, physiological stressors elicit a mitochondrial stress response that is important for maintenance of mitochondrial health and can improve cellular resilience as a hormetic action (5, 6).

Mitohormesis (mitochondrial hormesis) is considered as an adaptive stress response important for metabolic health. The concept of mitohormesis posits that perturbations across different domains of mitochondrial biology by diverse stressors lead to alterations in cytosolic and nuclear signaling which induce cytoprotective pathways leading to an increased stress resistance (6–8). These mitohormetic pathways can act in a cell-autonomous fashion to preserve cellular function and survival, but also lead to the induction of so called mitokines, i.e. mitochondrial or nuclear encoded, secreted proteins acting as endocrine hormones (9). Ultimately this can improve metabolic health, thereby affecting aging and longevity. Increasing evidence points to a mild mitochondrial uncoupling as an important trigger of mitohormesis to improve metabolic health (6, 10). Indeed, targeted mitochondrial uncoupling has long been considered an attractive strategy to regulate whole-body energy homeostasis and metabolic health for the treatment of obesity and associated metabolic disorders (11).

The systemic effects and underlying molecular mechanisms of mitochondrial uncoupling have been largely elucidated with the help of m*Ucp1*-transgenic (TG) mice (12, 13), with a skeletal muscle directed low expression level of UCP1, which is usually only expressed in brown adipocytes (14). This leads to a slightly compromised skeletal muscle efficiency of mitochondrial oxidative phosphorylation (OxPhos) due to increased uncoupling of the respiratory chain (15). However, despite decreased muscle mass and strength, TG mice display a healthy metabolic phenotype characterized by increased energy expenditure, reduced hepatic steatosis, induction of thermogenic adipocytes in white fat (browning), and improved glucose homeostasis, conferring resistance to the harmful effects of obesogenic diets and thereby increasing longevity (16–19). Mechanistically, we could show that the mild mitochondrial uncoupling-induced OxPhos inefficiency promotes the induction of the integrated stress response (ISR) and metabolic remodeling in skeletal muscle (13, 20). Interestingly, this includes changes in the pattern of oxidative protein damage, displaying both increased and decreased oxidative protein modifications, concomitant with an induction of the endogenous antioxidant defense system (20, 21). Activation of the ISR also induces gene expression and secretion of fibroblast growth factor 21 (FGF21) and growth and differentiation factor 15 (GDF15) as secreted mitokines. Ablation of either FGF21 or GDF15 revealed that, while neither are necessary for muscle cell autonomous adaptations, both are required for metabolic remodeling (12, 13, 22, 23). FGF21, on one hand, proofed to be involved in the induction of browning of white adipose tissue (22), and GDF15, on the other hand, was found to be necessary for the systemic metabolic adaptations by eliciting a daytime-restricted anorexia via a muscle-brain crosstalk (24), highlighting the importance of temporal dynamics in understanding metabolic adaptations to mitochondrial uncoupling (23, 25). Importantly, this aspect is rarely addressed in basic research, where analyses performed in mouse models are usually restricted to a single time point, most often in the morning, i.e. early resting phase, which corresponds to early nighttime when translated to humans. In particular, the temporal dynamics of ISR induction in response to physiological variation of tissue-specific energy demands are still unexplored.

In the present study, we performed in depth exploratory analyses to identify temporal signatures of both muscle cell-autonomous and systemic ISR in the context of endocrine myokine signaling, cellular redox balance, oxidative defense, and ferroptosis signature.

## 2 Materials and methods

### 2.1 Animals

Wildtype and m*Ucp1*-transgenic (TG) at 17-20 weeks (wks) of age with a C57BL/6J background were used for the experiments (17). Mice were kept group-housed in a 12-h dark: 12-h light regime and were fed a standard chow diet (Sniff) with *ad libitum* access. Physical activity and food intake were monitored in single-caged mice with the TSE PhenoMaster (TSE Systems) at 17 wks of age. After five days single-caged, 20-wks old mice were sacrificed via isoflurane narcosis and final heart puncture at six timepoints in 4-hour intervals. Muscle tissue was immediately dissected and freeze clamped before storage at -80°C. Further tissues were collected next and frozen at -80°C. Animal experiments were approved by the Ministry of Agriculture and Environment (State Brandenburg, Germany, permission number 2347-16-2020).

### 2.2 Plasma hormone and myokine analyses

Blood was collected at sacrifice through heart puncture in heparin tubes (#41.1503.005; Sarstedt), centrifuged at 9,000g for 10 min at 4°C, and plasma was stored at −80°C. Plasma corticosterone was measured with a Corticosterone ELISA kit (#ADI-900-097; Enzo). Active ghrelin, insulin, leptin and FGF21 were measured with a Meso-Scale Discovery (MSD) multiplex assay (MSD Instruments). GDF15 was quantified using the Mouse/Rat GDF-15 Quantikine ELISA Kit (#MGD150; Bio-Techne).

### 2.3 Gene expression analysis

A phenol-chloroform–based extraction using peqGOLD Trifast (#732-3314; VWR) was conducted to isolate RNA, which was followed by a DNase digestion (#EN0521; Thermo Fisher Scientific). cDNA synthesis was performed with the LunaScript RT SuperMix Kit (#E3010L; NEB). For quantitative real-time PCR (qPCR) analyses, 5 ng of cDNA, LUNA Universal Probe qPCR Mastermix (#M3004E; NEB), and 1.5 μM of primers in a total volume of 5 μl were used. Measurements were performed on a ViiA 7 Real-Time PCR System (Applied Biosystems). The following primers were used: *B2m*: 5′ CCCCACTGAGACTGATACATACGC 3′ (F), 5′ AGAAACTGGATTTGTAATTAAGCAGGTTC3′ (R), *Atf4*: 5′ GGAATGGCCGGCTATGG 3′ (F), 5′ TCCCGGAAAAGGCATCCT 3′ (R); *Atf5*: 5′CTACCCCTCCATTCCACTTTCC 3′ (F), 5′TTCTTGACTGGCTTCTCACTTGTG 3′ (R), *Chop*: 5′ AGAGTGGTCAGTGCGCAGC 3′ (F), 5′ CTCATTCTCCTGCTCCTTCTCC 3′ (R), *Fgf21*: 5′ GCTGCTGGAGGACGGTTACA 3′ (F), 5′ CACAGGTCCCCAGGATGTTG 3′ (R), *Gdf15*: 5′ GAGCTACGGGGTCGCTTC 3′ (F), 5′ GGGACCCCAATCTCACCT 3′ (R), *Ucp1* 5′ TGGAGGTGTGGCAGTATTC 3′ (F), 5′ AGCTCTGTACAGTTGATGATGAC 3′ (R).

### 2.4 Immunoblotting

25 mg of skeletal muscle (quadriceps) or interscapular BAT (iBAT) were homogenized in 450 μl RIPA buffer (50 mM Tris-HCl, 150 mM NaCl, 1 mM EDTA, 0,25% Na-Desoxycholate, 1% Triton X-100; pH 7.2) containing protease and phosphatase inhibitor cocktail (#A32959; Thermo Fisher Scientific) and 10 zirconium beads in a TissueLyser LT (Qiagen, USA) for 3 min at 50 Hz. Total protein content was measured with the DC Protein Assay Reagent (#500-0114; Bio-Rad) following manufacturer instructions. For electrophoresis, 15 μg of protein were loaded for every sample. After sufficient protein separation, semi-dry western blotting was performed according to manufacturer instructions using the Trans Blot TurboTM system (BioRad). Membranes were blocked for 1 h at RT in 5% milk powder in TBST and, after washing, incubated overnight at 4°C with the following antibodies and dilutions in 5% BSA in TBST: phospho-eIF2α (Ser51) (#3597, Cell Signaling Technology) diluted 1:1000, eIF2α (#3524, Cell Signaling Technology) diluted 1:1000, GPX1 (ab10842, abcam) diluted 1:5000, GPX4 (ab125066, abcam) diluted 1:5000, Ferritin Heavy Chain (ab183781, abcam) diluted 1:5000 and UCP1 (ab10983, abcam) diluted 1:2000. After washing with TBST, membranes were incubated in horseradish peroxidase-conjugated secondary antibodies diluted 1:20000 in 5% milk in TBST for 1 h at RT. Secondary antibodies used were anti-rabbit IgG (#7074, Cell Signaling Technology) or anti-mouse IgG (#7076, Cell Signaling Technology). Protein expression was analyzed with the software ImageJ. Unless otherwise stated, raw intensity values were normalized to the control group (WT, 10 am).

### 2.5 Enzymatic activity analysis

To obtain protein lysates, frozen muscle samples were homogenized in Tris buffer (100 mM, 300 mM KCl, 0.1% Triton X-100 with 0.1% protease inhibitor) using the TissueLyser II (Qiagen) for 2x 30 s at maximum speed. Afterwards, cellular debris was removed by centrifugation (14.000 x g, 4°C, 10 min) and protein concentration was measured by Bradford analysis (#500-0006, Bio-Rad).

Measurement of glutathione peroxidase (GPX) (26) and NAD(P)H quinone dehydrogenase 1 (NQO1) (27) activity was performed as described previously. Briefly, GPX activity was measured using a NADPH-dependent glutathione reductase coupled assay and NQO1 activity was conducted using a menadione-mediated reduction of 3-(4,5-dimethylthiazol-2-yl)-2,5-diphenyltetrazolium bromide (MTT). All enzymatic measurements were performed in triplicates using a microplate reader (Synergy H1) and were normalized to cellular protein concentration.

### 2.6 Plasma and skeletal muscle selenium and iron measurement

Selenium and iron concentrations were determined by Total Reflection X-ray Fluorescence spectrometry (TXRF), as reported before (28). In short, plasma was spiked with 1000 µg/l gallium (#16639, Sigma/Merck) as an internal standard, while muscle lysate based on RIPA buffer was spiked with 1 mg/l yttrium (#1198090100, Merck/Millipore). For quantification, plasma and muscle lysates spiked with internal standard were applied to non-siliconized and siliconized sample carriers, respectively, dried at 40°C, and measured for 1000 s in an X-ray fluorescence spectrometer (S4 T-STAR, Bruker). The trace element content of the muscle was normalized to the total protein content of the samples, determined with the DC protein assay reagent (#500-0114; Bio-Rad).

### 2.7 Skeletal muscle MDA measurement

Malondialdehyde (MDA) was quantified by high-performance liquid chromatography (HPLC) with fluorescence detection in 10 mg of frozen muscle tissue homogenized in 100 μl cold RIPA buffer (pH 7,4). Briefly, homogenates were derivatized with thiobarbituric acid and further proceeded as described previously (29). Total MDA was normalized to protein content measured by a Bradford Assay.

### 2.8 Statistical analysis

Statistical analyses were performed using GraphPad Prism 9 (GraphPad Software, San Diego, CA, USA). Differential oscillation within 24 hours and difference between the genotypes were analyzed using two-way ANOVA with the Geisser-Greenhouse correction (*P*-values are shown for each graph).

## 3 Results

### 3.1 Systemic regulation of energy homeostasis

Ectopic expression of UCP1 in TG mice leads to mRNA concentrations in skeletal muscle that are comparable to interscapular brown adipose tissue (iBAT) (**Supplementary Figures 1A–D**) while UCP1 protein abundance was around 4-5 times higher in iBAT compared to skeletal muscle (**Supplementary Figures 1E–H**). Of note, skeletal muscle of wildtype (WT) littermates showed neither gene nor protein expression of UCP1.

**Figure 1:**
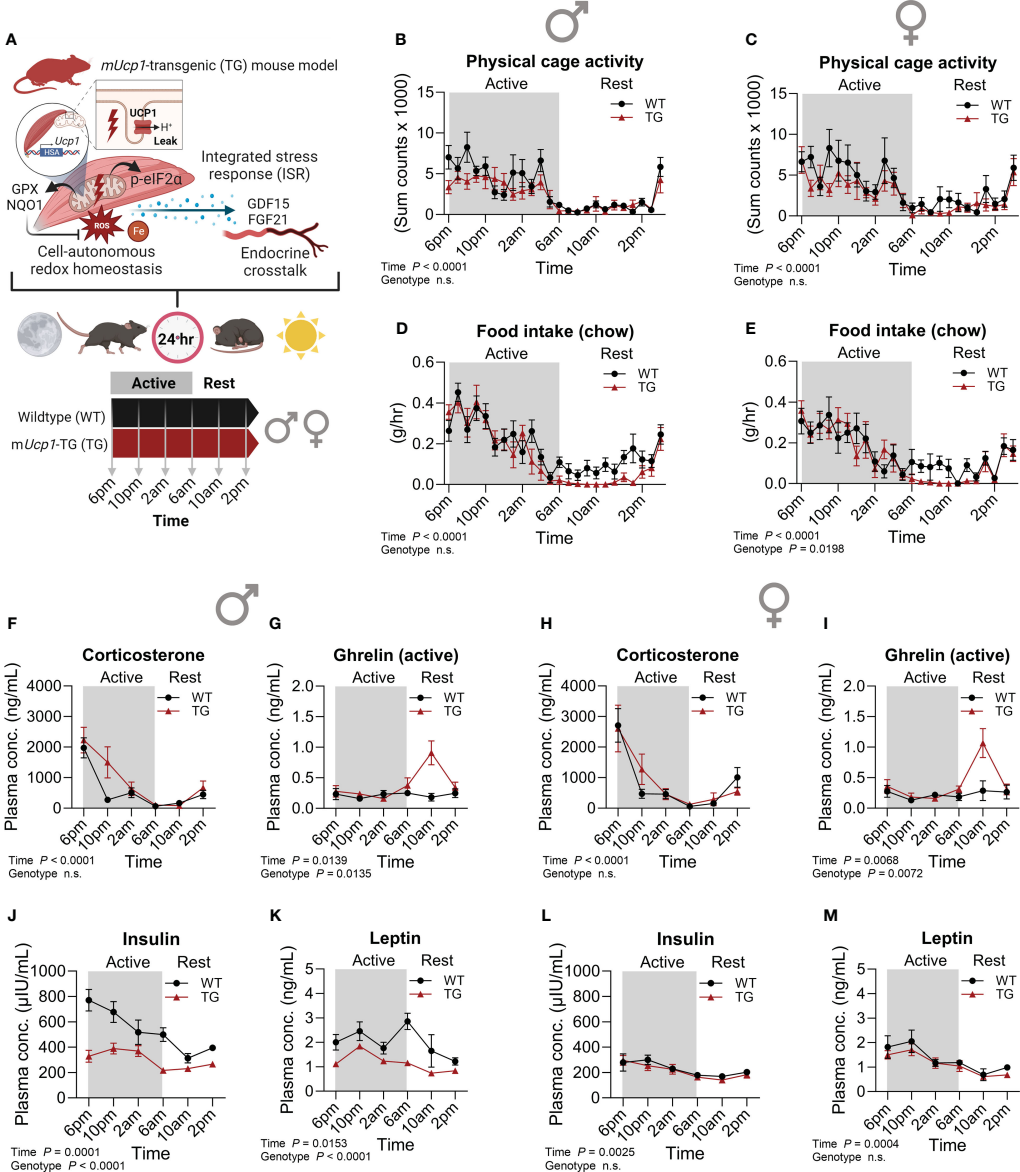
Metabolic phenotype and temporal dynamics of circulating hormones. **(A)** Schematic representation of the study design. **(B, C)** Physical activity and **(D, E)** food intake over a 24-hour period in male and female mice, respectively (n=7-10 per genotype and sex). Plasma **(F, H)** corticosterone, **(G, I)** ghrelin (active), **(J, L)** insulin, and **(K, M)** leptin in male and female mice, respectively (n=5-7 per genotype, sex and timepoint). WT, wildtype; TG, m*Ucp1*-transgenic. Samples were harvested during a 24-hour period in 4-hour intervals. Data were analyzed using a two-way ANOVA with the Geisser-Greenhouse correction.

Mice exhibit circadian rhythms with diurnal/nocturnal pattern of locomotor activity powered by contraction of skeletal muscle which is controlled by muscle circadian clock and energy metabolism (30, 31). Therefore, our first objective was to establish the diurnal dynamics of energy homeostasis and key metabolic hormones of TG mice in comparison to WT littermates (**Figure 1A**). Male and female mice were sacrificed at 4-hour intervals throughout the day-night cycle with collection of plasma and skeletal muscle samples for subsequent analyses. Analyses of diurnal activity and feeding behavior are consistent with previous studies (23, 24) and indicate that both male and female TG mice exhibit normal physical activity behavior within their cages (**Figures 1B, C**; **Supplementary Figures 1I, K**). However, during the resting phase, a distinct anorexia was observed in both male and female TG mice (**Figures 1D, E**; **Supplementary Figures 1J, L**), which, as shown by our previous studies, is governed by the GDF15-GFRAL axis (23, 24). Notably, this diurnal anorexic behavior was not associated with altered dynamics of circulating corticosterone (**Figures 1F, H**) indicating minor effects of skeletal muscle uncoupling on the systemic stress response via hypothalamic–pituitary–adrenal axis. However, both male and female TG mice showed a peak of active ghrelin 4h after lights on in the resting phase (**Figures 1G, I**), reflecting a hunger signal from the stomach triggered by suppression of food intake. Of note, diurnal profiles of corticosterone and ghrelin were similar in male and female WT as well as TG mice. In contrast, while male TG mice displayed robust lower insulin and leptin levels in plasma during the active and resting phase (**Figures 1J, K**), these effects were fully absent in female TG mice across the 24-hour cycle (**Figures 1L, M**), a fact that could be due to overall higher circulating insulin and leptin in WT males compared to females. As shown before, TG mice displayed a reduced body weight (**Supplementary Figures 1M, N**) which is mainly based on a reduced lean body mass and skeletal muscle atrophy (15, 16).

Taken together, our findings suggest that in TG mice the anorexia during rest is indeed not a consequence of insulin or leptin signaling, but rather driving their alterations, implying the involvement of other, possibly sex-specific metabolic adaptations and signaling mechanisms. Nevertheless, our previous findings show a sex-independent pattern of hypothalamic gene expression of TG mice in the early resting phase reflecting a state of negative energy balance with increased appetite signaling resembling an anorexia nervosa-like phenotype that we demonstrated to be controlled by the GDF15-GFRAL muscle-brain axis (24).

### 3.2 Cell-autonomous and systemic integrated stress response pathways in TG mice

**Figure 2:**
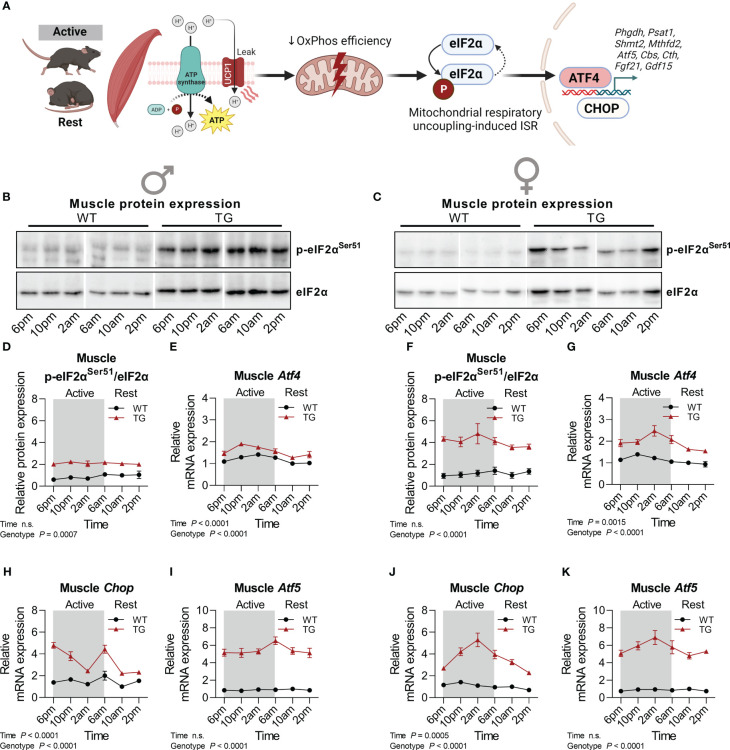
Temporal fluctuations of muscle mitochondrial ISR induction. **(A)** Graphical representation of the activation of the integrated stress response (ISR) pathway by mitochondrial stress in m*Ucp1*-transgenic mice. **(B, C)** Representative immunoblots and **(D, F)** quantifications of p-eIF2α^Ser51^ normalized to total eIF2α in skeletal muscle of male and female mice, respectively (n=3-4 per genotype, sex, and timepoint). **(E, G)** Relative mRNA expression of *Atf4*, **(H, J)** *Chop*, and **(I, K)** *Atf5* in skeletal muscle of male and female mice, respectively (n=6-8 per genotype, sex, and timepoint). WT, wildtype; TG, m*Ucp1*-transgenic. Samples were harvested during a 24-hour period in 4-hour intervals. Data were analyzed using a two-way ANOVA with the Geisser-Greenhouse correction.

The ISR has been proposed as the main molecular pathway that promotes cell-autonomous transcriptional adaptations and metabolic remodeling (**Figure 2A**), including the induction of the endocrine acting cytokines GDF15 and FGF21 under various manifestations of mitochondrial dysfunction in different organs (32). As a central ISR mediator, total protein abundance and phosphorylation of eukaryotic translation initiation factor 2α (eIF2α) were highly increased in skeletal muscle of male and female TG mice, while no diurnal variations were observed (**Figures 2B–D, F**). However, downstream transcriptional activation of activating transcription factor 4 (*Atf4*), *Atf5* and C/EBP homologous protein (*Chop*) revealed temporal variations with highest levels in the late active phase, that were more pronounced and consistent in female TG mice, while in WT mice no diurnal oscillations were apparent (**Figures 2E, G, H–K**).

**Figure 3:**
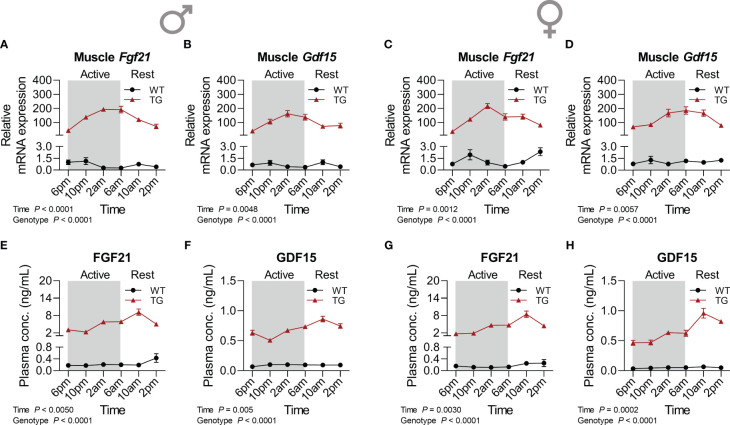
Time-dependent pattern of myokine induction. **(A, C)** Relative mRNA expression of *Fgf21* and **(B, D)** *Gdf15* in skeletal muscle of male and female mice, respectively (n=6-8 per genotype, sex, and timepoint). **(E, G)** FGF21 and **(F, H)** GDF15 concentrations in plasma of male and female mice, respectively (n=6-9 per genotype, sex, and timepoint). WT, wildtype; TG, m*Ucp1*-transgenic. Samples were harvested during a 24-hour period in 4-hour intervals. Data were analyzed using a two-way ANOVA with the Geisser-Greenhouse correction.

We have previously demonstrated the induction of FGF21 and GDF15 as non-cell-autonomous, systemic effects of muscle mitochondrial uncoupling (12, 23). Here we analyzed in detail their diurnal transcriptional and circulating profile (**Figure 3**). Strikingly, muscle mRNA expression of both *Fgf21* (**Figures 3A, C**) and *Gdf15* (**Figures 3B, D**) showed strong diurnal oscillations for both cytokines and both sexes in TG mice, which corresponded to strong temporal dynamics in plasma GDF15 and FGF21 levels (**Figures 1E–H**) with a delay of approximately 4 hours between peak mRNA expression in muscle in the late active phase and circulating protein peak in the early resting phase. Taken together, our data uncover a diurnal regulation at the transcriptional level of key ISR target genes under mitochondrial uncoupling.

### 3.3 Redox homeostasis promoted by skeletal muscle mitochondrial uncoupling is regulated in a diurnal manner

**Figure 4:**
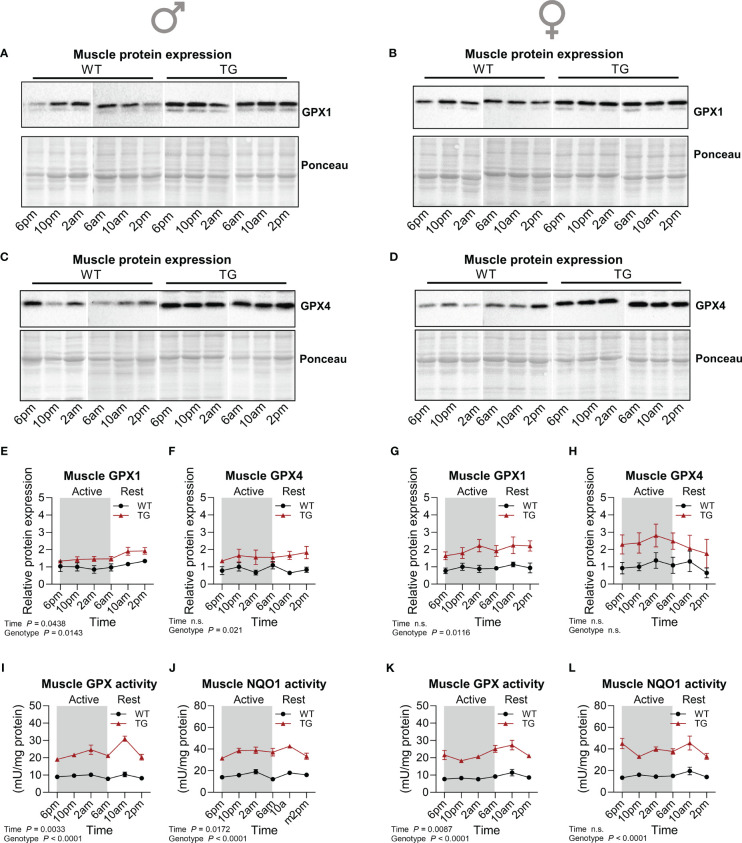
Temporal dynamics of skeletal muscle antioxidant response. **(A-H)** Representative immunoblots and quantifications of GPX1 **(A, B, E, G)** and GPX4 **(C, D, F, H)** normalized to Ponceau staining in skeletal muscle of male and female mice, respectively (n=3-4 per genotype, sex, and timepoint). **(I, K)** GPX and **(J, L)** NQO1 activity in skeletal muscle of male and female mice, respectively (n=4-5 per genotype, sex and timepoint). WT, wildtype; TG, m*Ucp1*-transgenic. Samples were harvested during a 24-hour period in 4-hour intervals. Data were analyzed using a two-way ANOVA with the Geisser-Greenhouse correction.

One of the mechanisms proposed for the induction of adaptive endoplasmic reticulum (ER) stress and the ISR in TG mice is the generation of reactive oxygen species (ROS) and thus, oxidative stress (20). Central for cellular oxidative defense and regulation of cellular redox homeostasis are glutathione peroxidases (GPX), that reduce hydroperoxides using reduced glutathione (GSH) as a substrate (33). We have previously shown that GPX activity and GPX1 protein content were increased (34) while there were no differences in oxidized glutathione (GSSG) or total GSH in muscle of TG compared to WT mice (21). This suggests that TG mice need to upregulate their oxidative defense mechanisms in order to maintain normal redox homeostasis. Here we found that muscle GPX1 and GPX4 protein content was slightly increased in TG mice with minor diurnal variations of GPX1 in male TG mice only (**Figures 4A–H**). GPX1 and 4 are selenoproteins, and utilization of selenium is essential for the function of GPX4 (35). Plasma and muscle total selenium content were elevated in both male and female TG mice and regulated in a temporal manner (**Supplementary Figures 2A–D**). In contrast to GPX4, total GPX activity, representing mainly GPX1 (36), is more sensitive towards changes in the selenium supply of the cells. Thus, high muscle selenium concentrations of TG mice around 2am could cause highly significant diurnal variations in both male and female mice with highest GPX activities found in the early resting phase and consistently elevated levels in TG compared to WT (**Figures 4I, K**). In addition, the activity of NAD(P)H quinone oxidoreductase 1 (NQO1), a target of nuclear factor erythroid 2–related factor 2 (Nrf2), and another important redox enzyme, was highly increased in skeletal muscle of TG mice during the 24-hour cycle, confirming previous findings (34) (**Figures 4J, L**). Interestingly, it appeared to have a mild diurnal variation similar to that of GPX in male mice (**Figure 4J**), while in female mice it presented irregular variations over the 24-hour cycle (**Figure 4L**).

### 3.4 Lipid peroxidation and markers of ferroptosis defense are upregulated during skeletal muscle mitochondrial uncoupling

**Figure 5:**
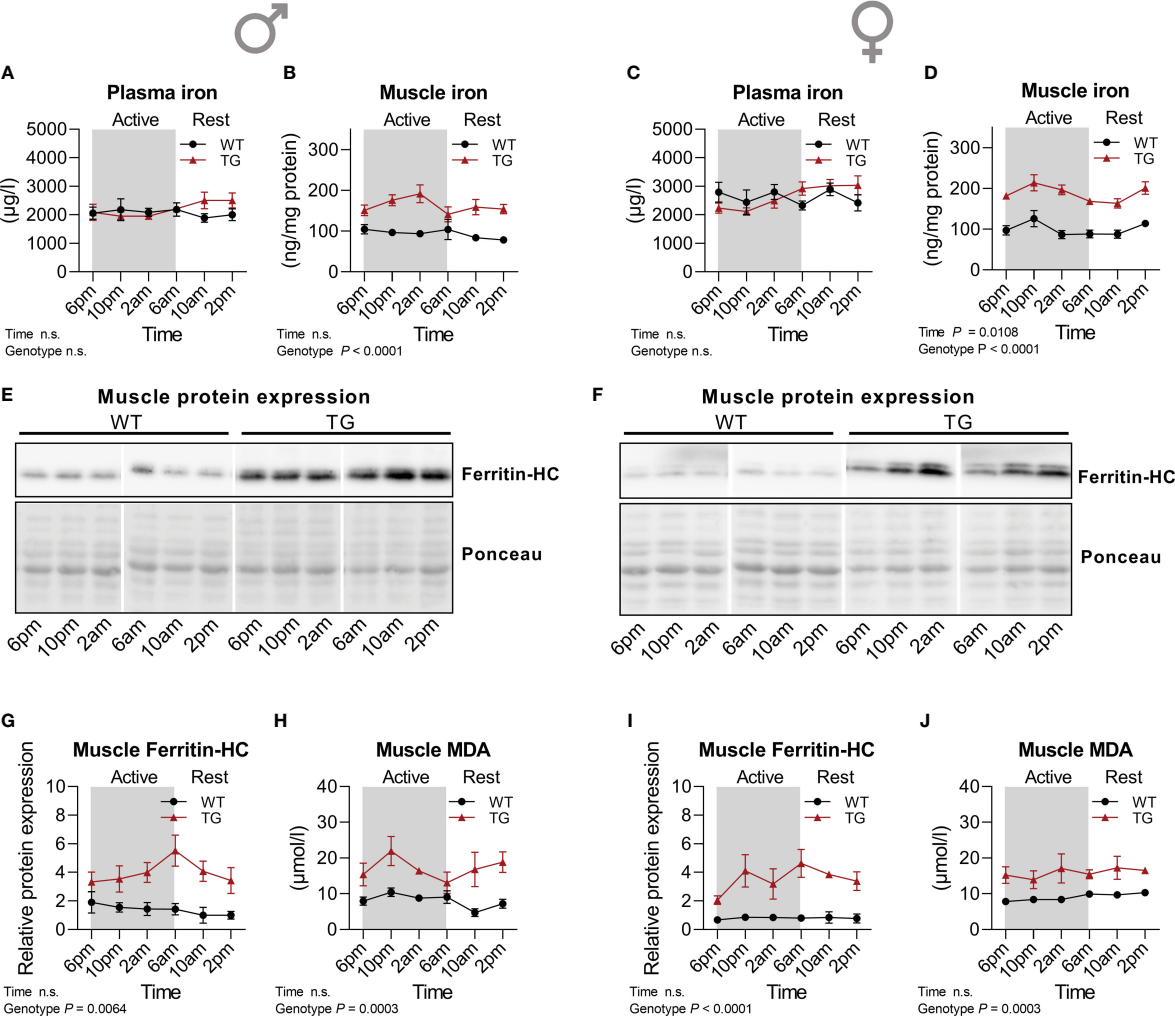
Time-dependent regulation of ferroptosis. **(A, C)** Plasma (n=7-8 per genotype, sex and timepoint) and **(B, D)** skeletal muscle (n=4-5 per genotype, sex and timepoint) iron concentration of male and female mice, respectively. **(E, F)** Representative immunoblots and **(G, I)** quantifications of Ferritin Heavy Chain (Ferritin-HC) normalized to Ponceau staining in skeletal muscle of male and female mice, respectively (n=4-5 per genotype, sex, and timepoint). **(H, J)** Skeletal muscle MDA content of male and female mice, respectively (n=5 per genotype, sex, and timepoint). WT, wildtype; TG, m*Ucp1*-transgenic. Samples were harvested during a 24-hour period in 4-hour intervals. Data were analyzed using a two-way ANOVA with the Geisser-Greenhouse correction.

An important consequence of oxidative stress and ROS accumulation is lipid peroxidation, which results from the reaction of ROS with polyunsaturated fatty acids (PUFAs) in cell membranes, leading to changes in membrane composition which affects fluidity and permeability and eventually leads to an iron-dependent type of cell death known as ferroptosis (37). Ferroptosis is thus characterized by the accumulation of lipid peroxides (LOOH) (38). We have previously reported an increased lipid oxidation as evident by elevated malondialdehyde (MDA) levels in skeletal muscle of TG mice concomitant with increased antioxidative defense mechanisms such as increased catalase activity (20), and here we show an increased muscle GPX activity that is regulated in a diurnal manner. Thus, we hypothesized that a diurnal regulation of the antioxidant system in TG mice skeletal muscle might lead to diurnal variations in the molecular mechanisms involved in ferroptosis. Analysis of circulating and muscle iron levels revealed that in both male and female TG mice muscle but not circulating total iron was increased compared to WT, while no pronounced diurnal variations could be detected (**Figures 5A–D**). One of the most relevant proteins involved in the regulation of ferroptosis is ferritin, an iron storage protein complex (38). Ferritin can inhibit ferroptosis by the ferroxidase activity of its heavy chain (39). Protein expression of ferritin heavy chain (Ferritin-HC) was highly induced in skeletal muscle of both male and female TG mice compared to WT mice, while no apparent diurnal variations were observed in either genotype (**Figures 5E–G, I**). Similarly, muscle MDA levels were constantly increased in TG mice (**Figures 5H, J**). These data indicate an induction of defense mechanisms to limit muscle ferroptosis which results from the increased oxidative stress induced by muscle uncoupling-induced hypermetabolism. Finally, by a re-analysis of our previously published transcriptome dataset (GSE45991 (13)), we here show an increased expression of ferroptosis protecting and decreased expression of ferroptosis promoting genes in skeletal muscle of male TG mice (**Supplementary Figures 2E, F**). TG mice show no major morphological difference and functional muscle impairment compared to WT muscle (15). We thus hypothesize that the increased antioxidative and ferroptosis defense mechanisms in TG mice serve to protect their muscle integrity.

## 4 Discussion

Given the importance of circadian rhythms and diurnal variations in mitochondrial function and regulation, we aimed to identify the temporal signature of muscle respiratory uncoupling-induced stress adaptations using the well characterized TG mouse model with ectopic expression of UCP1 in skeletal muscle (6). The role of UCP1 and mitochondrial uncoupling in redox homeostasis is quite complex. On one hand there is ample evidence that mitochondrial uncoupling prevents oxidative stress by limiting ROS production through a decrease in mitochondrial membrane potential. In isolated mitochondria from skeletal muscle of TG mice we have shown that UCP1 displays native uncoupling behavior and mitigates ROS (superoxide) production (40). On the other hand, other studies suggested that UCP1 can augment ROS production indirectly by increasing mitochondrial fuel metabolism (reviewed by (41)). We previously found that the ectopic expression of UCP1 in muscle mitochondria leads to an increase in oxidative stress that in turn increases endogenous antioxidant defense systems such as catalase, GPX, and NQO1 in TG mice (20, 34). Together with our findings of a consistently increased ferroptosis signature this suggests that the uncoupling-induced hypermetabolism in muscle leads to an increased ROS production.

**Figure 6:**
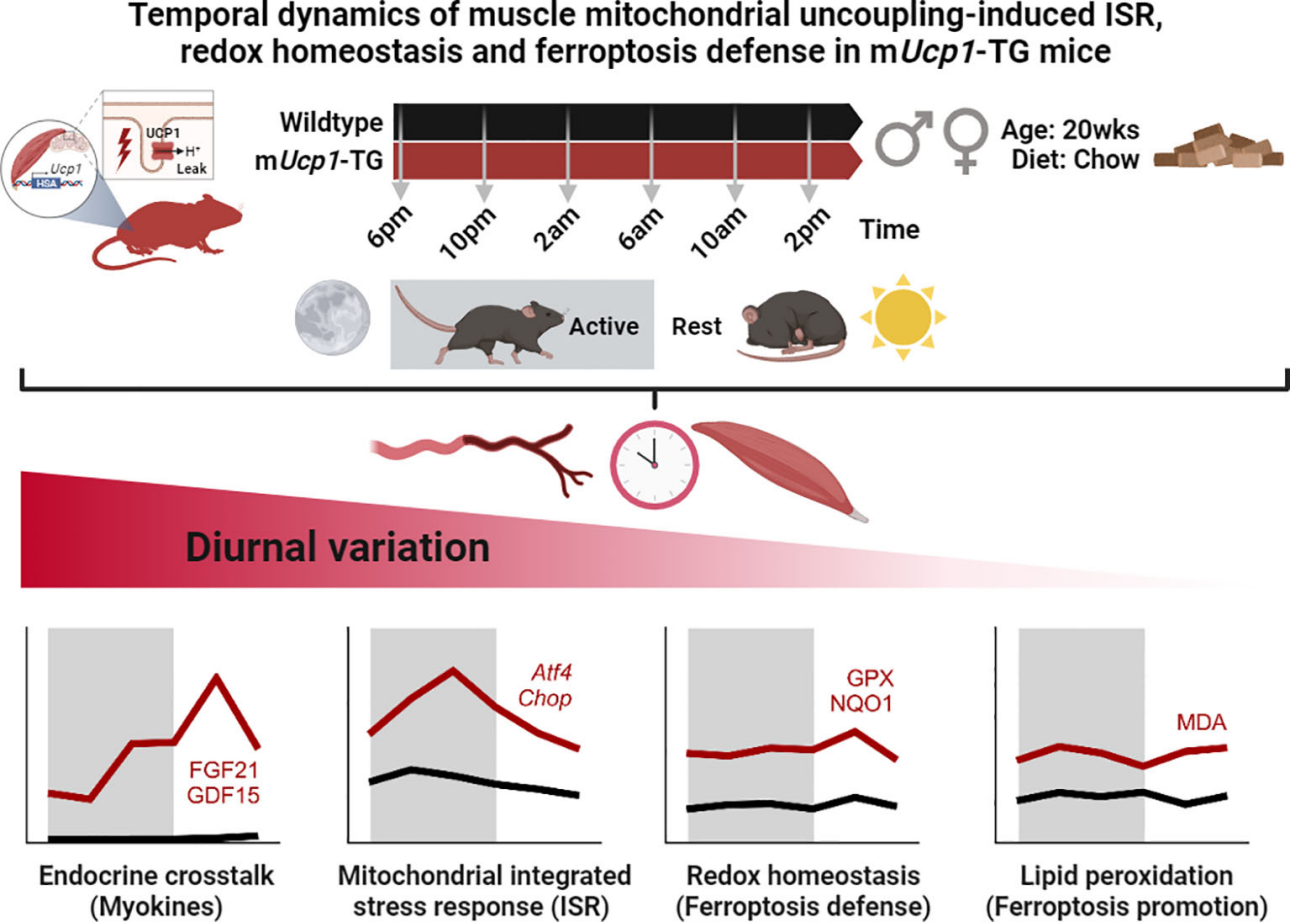
Graphical abstract and summary of the main results. While endocrine stress induced myokines, ISR, redox defense, and lipid peroxidation were consistently elevated in TG muscle, they differed in the pattern and amplitude of their diurnal variation.

Importantly, we here demonstrate a temporal signature of both cell-autonomous and systemic ISR in the context of endocrine myokine signaling, redox balance, and oxidative defense against ferroptosis in skeletal muscle of TG mice. Our observations of diurnal variations in redox homeostasis and oxidative defense mechanisms emphasize the importance of circadian control in maintaining cellular and systemic homeostasis. Finally, we identify that these diurnal adaptations to uncoupling-induced oxidative stress appear to be dissociated from a time-independent lipid peroxidation signature as summarized in **Figure 6**.

Over the past decade, the association of impairments across different domains of mitochondrial biology with ISR induction has been demonstrated in several mouse models (32), but the interaction of cellular and systemic ISR mechanisms remains poorly understood. Moreover, there is increasing awareness that the disruption of our natural biological rhythms strongly contributes to the development of metabolic diseases such as obesity and type 2 diabetes that result from a metabolic dysregulation and disturbed energy homeostasis (42, 43). Here we show a temporal coupling of cell-autonomous ISR with endocrine signaling through myokines, further suggesting a complex coordination between tissue-specific responses and whole-body adaptations which, as the present study highlights, seems to be affected by sex. While cellular and systemic ISR signaling as well as antioxidant mechanisms present a sex-independent pattern, endocrine signaling of the metabolic hormones insulin and leptin appear to be disrupted in male but not in female TG mice (**Figures 1J–M**), possibly responding to an action of estrogen as a key regulator of energy metabolism in females (44). Importantly, numerous studies have demonstrated elevated levels of serum FGF21 and GDF15 in patients with mitochondrial disease (MD) characterized by myopathy (45–47), but also in age-related diseases (48). However, as a standard procedure in clinical practice, blood samples from patients or subjects are usually drawn in the morning after overnight fasting which, as the present study highlights, corresponds to the time of the early active phase in mice and is associated with the lowest circulating levels of FGF21 and GDF15. Hence, performing multiple sample collections throughout the day has the potential to enhance the diagnostic value of early biomarker analysis in patients who remain undiagnosed so far, and will validate possible therapeutic benefits of interventions targeting mitochondrial disorders.

Furthermore, our study suggests that the ISR induced upon respiratory uncoupling in skeletal muscle, which appears to be regulated in a diurnal manner, supports the oxidative defense against ferroptosis. It has been suggested that the systemic ferroptotic response might be controlled by circadian rhythm and clock genes (38). However, the origin of our here observed uncoupling-driven cell autonomous as well as systemic oscillations in TG mice remains, so far, unknown. It is tempting to hypothesize that these oscillations might emerge from activity-dependent oscillations in ROS production, which would accumulate during the active phase in the metabolically compromised skeletal muscle of TG mice leading to a time-dependent oxidative stress response as our results suggest. Nevertheless, it is well accepted that the molecular machinery of the circadian clock directly controls mitochondrial function, including mitochondrial respiration (4). It can thus not be excluded that in the skeletal muscle of TG mice, clock-controlled oscillations in mitochondrial function are amplified as a result of an increased oxidative stress. In heart muscle, recent discoveries have shown that OxPhos impairments predispose cardiomyocytes to ferroptosis (49), although important questions concerning tissue-specificity, timing of the cellular response as well as potential temporal dynamics and quantitative aspects of defects in different functions of mitochondria and ferroptosis defense remain unanswered (50).

We previously showed that the activity of aconitase, a ROS-sensitive iron-sulfur protein that converts citrate to isocitrate and contributes to cellular iron homeostasis (51), as well as products of lipid peroxidation are increased in muscle of male TG mice (20, 21). Ferroptosis, a form of cell death driven by the accumulation of iron-dependent lipid peroxidation (38), has been suggested as a key contributing factor to various skeletal muscle diseases (37), and mitochondrial function is pivotal for both induction of and protection from ferroptosis (50, 52). The selenoproteins GPX1 and GPX2 have been described to reduce soluble hydroperoxides, while GPX4 exclusively reduces complex lipid hydroperoxides (phosphatidylcholine hydroperoxide), thus protecting cells from lipid peroxidation and ferroptosis (36). Strikingly, we here show a robust induction of both GPX1 and GPX4 protein expression during the active and resting phase together with a minor but significant diurnal variation of increased total GPX-activity in muscle of TG mice. Moreover, although the direct role of the NRF-target NQO1 in ferroptosis regulation under physiological conditions remains negligible (53), we have previously shown the biological relevance of the NRF2-dependent oxidative stress defense under muscle mitochondrial OxPhos impairments (34). While recent *in-vitro* data provide new insight into the importance of the ubiquitin-proteasome system (UPS) in ferroptosis (54), the role of UPS in skeletal muscle upon chronic mitochondrial stress remains to be elucidated.

The induction of antioxidative defense mechanisms against ferroptosis in response to a disturbed mitochondrial OxPhos function highlights the importance of maintaining redox balance and iron homeostasis as an adaptive, prosurvival mechanism to prevent myofiber damage and progression of a mitochondrial myopathy. In prior studies, we demonstrated that despite chronic respiratory uncoupling-induced OxPhos inefficiency in the muscles of TG mice, there were no significant impairments in muscle physiology or morphology. Histological analysis revealed reduced fiber size but no noticeable degenerative changes in skeletal muscle morphology, such as central nuclei, misalignment of Z-lines, or abnormal mitochondria (12). Overall, we have shown that muscle function and integrity is preserved in TG mice which present as a model of healthy aging despite their reduced muscle mass and strength (15, 16). Interestingly, there is ample evidence linking a slightly increased mitochondrial uncoupling to an increased lifespan in rodents (16), which we suggested to be, at least partly, due to cell-autonomous and systemic mitohormetic adaptations (6). The fact that TG mice present a higher skeletal muscle iron status with unchanged plasma iron concentrations could indicate a higher iron uptake, which could be ultimately due to the previously reported higher OxPhos levels in TG mice and a fast (glycolytic) to slow (oxidative) muscle fiber type switch (15, 20, 55). Thus, high ferritin levels in TG mice could represent an adaptation to a more intensive mitochondrial/iron metabolism in the skeletal muscle. The role of ferritin as an iron storage protein in limiting ferroptosis provides valuable insights into potential therapeutic strategies for conditions involving mitochondrial dysfunction and oxidative stress.

Interestingly, a recent study revealed that the ISR, which is highly induced in skeletal muscle of TG mice (12, 13) promotes ATF4-dependent activation of NRF2 via a gamma-glutamylcyclotransferase 1 (CHAC1)-mediated GSH depletion, leading to NRF2 stabilization (56). Interestingly, we found *Chac1* to be upregulated in male TG mice (see published microarray dataset GSE45991, **Supplementary Figure 2E**) (13). Additionally, we observed increased gene expression of *Slc7a11*, *Slc3a2*, *Gpx4*, and *Scd1* (**Supplementary Figure 2E**), all of which contribute to resistance against ferroptosis (57). It is well-accepted that peroxidation of PUFAs plays a pivotal role in ferroptosis promotion, while the activity of PUFAs in ferroptosis is suggested to be competitively affected by mono-unsaturated fatty acids (MUFAs, e.g., oleic acid) to drive ferroptosis resistance, which involves stearoyl-CoA desaturase (SCD1) (58). Further, lipid peroxidation is regulated by system xCT (also known as X_C_^−^), which consists of a light-chain subunit (SLC7A11/xCT) and a heavy-chain subunit (SLC3A2) to transport cysteine, an important precursor for GSH synthesis, into the cell (59). In contrast, several lipid metabolic enzymes, including acyl-coenzyme A (CoA) synthetase long-chain family member 4 (ACSL4) and lyso-phosphatidylcholine acyltransferase 3 (LPCAT3), contribute to PUFA-phospholipid synthesis and remodeling, influencing cellular lipid composition to promote ferroptosis (60, 61). Notably, the expression of *Ascl4* and *Lpcat3*, markers for PUFA-phospholipid biosynthesis and ferroptosis, was downregulated in TG muscle (**Supplementary Figure 2E**), in line with an unpublished lipidomic analysis in muscle samples of 20-week-old TG mice that revealed lower total PUFA(n-6) levels (data not shown). Altogether, this supports the notion of an adaptive, anti-ferroptotic transcriptional remodeling in response to chronic mild mitochondrial uncoupling, consistent with preserved muscle integrity and homeostasis.

In conclusion, our study sheds new light on the intricate regulatory mechanisms involved in cellular and mitochondrial integrated stress responses and metabolic adaptations driven by muscle respiratory uncoupling. These findings have implications for translational nutritional strategies and diagnostic biomarker analysis, as the temporal dynamics of cellular stress responses need to be considered when studying and treating metabolic disorders and related diseases.

## Data availability statement

The raw data supporting the conclusions of this article will be made available by the authors, without undue reservation.

## Ethics statement

The animal study was approved by Ministry of Agriculture and Environment (State Brandenburg, Germany, permission number 2347-16- 2020). The study was conducted in accordance with the local legislation and institutional requirements.

## Author contributions

CG: Conceptualization, Data curation, Formal Analysis, Funding acquisition, Investigation, Methodology, Project administration, Resources, Validation, Visualization, Writing – original draft, Writing – review & editing. AL: Investigation, Methodology, Writing – review & editing. KL: Investigation, Methodology, Writing – review & editing. MS: Investigation, Methodology, Writing – review & editing. DW: Investigation, Methodology, Writing – review & editing. TG: Funding acquisition, Writing – review & editing. AK: Resources, Writing – review & editing. SK: Conceptualization, Funding acquisition, Resources, Supervision, Writing – original draft, Writing – review & editing. MO: Conceptualization, Data curation, Formal Analysis, Funding acquisition, Investigation, Methodology, Project administration, Resources, Supervision, Validation, Visualization, Writing – original draft, Writing – review & editing.

## Supplementary material

Supplementary Figure 1 Phenotypic characterization of m*Ucp1*-transgenic mice. **(A-D)** *Ucp1* mRNA expression in interscapular BAT (iBAT) and skeletal muscle represented as raw ct values **(A, C)** and Δct values using *B2m* as housekeeping gene **(B, D)** in male and female mice, respectively (n=6 per genotype, sex and tissue). **(E, G)** Representative immunoblots and **(F, H)** quantification of UCP1 expression in iBAT and muscle in male and female mice, respectively. Relative protein expression was calculated normalizing raw intensity values to iBAT WT samples (n= 5-6 per genotype, sex and tissue). **(I-L)** Quantification of total active- *versus* inactive phase physical activity **(I, K)** and chow intake **(J, L)** in male and female mice, respectively (n=7-10 per genotype and sex). **(M, N)** Body weight of mice sacrificed during a 24-hour period in 4-hour intervals corresponding to (n=7-8 per genotype and sex). WT, wildtype; TG, m*Ucp1*-transgenic. Data were analyzed using a one-way ANOVA **(B, D, F, H, I-L)** or two-way ANOVA with the Geisser-Greenhouse correction (m, n). Unless otherwise indicated, data correspond to mice sacrificed at 10 am.

Supplementary Figure 2 Temporal variation of plasma and muscle selenium. **(A, C)** Plasma (n=7-8 per genotype, sex and timepoint) and **(B, D)** skeletal muscle (n=4-5 per genotype, sex and timepoint) selenium concentration in male and female mice, respectively. Samples were harvested during a 24-hour period in 4-hour intervals. WT, wildtype; TG, m*Ucp1*-transgenic. Data were analyzed using a two-way ANOVA with the Geisser-Greenhouse correction. **(E)** Heat map of ferroptosis related genes extracted from a published microarray analysis (GSE45991) of skeletal muscle in male TG mice collected at 10am. **(F)** Suggested mechanism of oxidative defense and ferroptosis signature in skeletal muscle of TG mice. Genes/proteins shown in red are upregulated while those shown in blue are downregulated in TG muscle compared to WT.

## References

[1] Monzel AS Enriquez JA Picard M. Multifaceted mitochondria: moving mitochondrial science beyond function and dysfunction. Nat Metab (2023) 5:546–62. doi: 10.1038/s42255-023-00783-1 PMC10427836 37100996

[2] Picard M Shirihai OS. Mitochondrial signal transduction. Cell Metab (2022) 34:1620–53. doi: 10.1016/j.cmet.2022.10.008 PMC9692202 36323233

[3] de Goede P Wefers J Brombacher EC Schrauwen P Kalsbeek A. Circadian rhythms in mitochondrial respiration. J Mol Endocrinol (2018) 60:R115–30. doi: 10.1530/JME-17-0196 PMC5854864 29378772

[4] Manella G Asher G. The circadian nature of mitochondrial biology. Front Endocrinol (Lausanne) (2016) 7:162. doi: 10.3389/fendo.2016.00162 28066327 PMC5165042

[5] Casanova A Wevers A Navarro-Ledesma S Pruimboom L. Mitochondria: It is all about energy. Front Physiol (2023) 14:1114231. doi: 10.3389/fphys.2023.1114231 37179826 PMC10167337

[6] Klaus S Ost M. Mitochondrial uncoupling and longevity - A role for mitokines? Exp Gerontol (2020) 130:110796. doi: 10.1016/j.exger.2019.110796 31786315

[7] Yun J Finkel T. Mitohormesis. Cell Metab (2014) 19:757–66. doi: 10.1016/j.cmet.2014.01.011 PMC4016106 24561260

[8] Ristow M Schmeisser K. Mitohormesis: promoting health and lifespan by increased levels of reactive oxygen species (ROS). Dose Response (2014) 12:288–341. doi: 10.2203/dose-response.13-035.Ristow 24910588 PMC4036400

[9] Merry TL Chan A Woodhead JST Reynolds JC Kumagai H Kim SJ. Mitochondrial-derived peptides in energy metabolism. Am J Physiol Endocrinol Metab (2020) 319:E659–66. doi: 10.1152/ajpendo.00249.2020 PMC7750512 32776825

[10] Demine S Renard P Arnould T. Mitochondrial uncoupling: A key controller of biological processes in physiology and diseases. Cells (2019) 8(8):795. doi: 10.3390/cells8080795 31366145 PMC6721602

[11] Ost M Keipert S Klaus S. Targeted mitochondrial uncoupling beyond UCP1 - The fine line between death and metabolic health. Biochimie (2017) 134:77–85. doi: 10.1016/j.biochi.2016.11.013 27916644

[12] Keipert S Ost M Johann K Imber F Jastroch M van Schothorst EM. Skeletal muscle mitochondrial uncoupling drives endocrine cross-talk through the induction of FGF21 as a myokine. Am J Physiol Endocrinol Metab (2014) 306:E469–82. doi: 10.1152/ajpendo.00330.2013 24347058

[13] Ost M Keipert S van Schothorst EM Donner V van der Stelt I Kipp AP. Muscle mitohormesis promotes cellular survival via serine/glycine pathway flux. FASEB J (2015) 29:1314–28. doi: 10.1096/fj.14-261503 25491309

[14] Klaus S Casteilla L Bouillaud F Ricquier D. The uncoupling protein UCP: a membraneous mitochondrial ion carrier exclusively expressed in brown adipose tissue. Int J Biochem (1991) 23:791–801. doi: 10.1016/0020-711X(91)90062-R 1773883

[15] Ost M Werner F Dokas J Klaus S Voigt A. Activation of AMPKalpha2 is not crucial for mitochondrial uncoupling-induced metabolic effects but required to maintain skeletal muscle integrity. PloS One (2014) 9:e94689. doi: 10.1371/journal.pone.0094689 24732703 PMC3986237

[16] Keipert S Voigt A Klaus S. Dietary effects on body composition, glucose metabolism, and longevity are modulated by skeletal muscle mitochondrial uncoupling in mice. Aging Cell (2011) 10:122–36. doi: 10.1111/j.1474-9726.2010.00648.x PMC3042149 21070590

[17] Klaus S Rudolph B Dohrmann C Wehr R. Expression of uncoupling protein 1 in skeletal muscle decreases muscle energy efficiency and affects thermoregulation and substrate oxidation. Physiol Genomics (2005) 21:193–200. doi: 10.1152/physiolgenomics.00299.2004 15687481

[18] Katterle Y Keipert S Hof J Klaus S. Dissociation of obesity and insulin resistance in transgenic mice with skeletal muscle expression of uncoupling protein 1. Physiol Genomics (2008) 32:352–9. doi: 10.1152/physiolgenomics.00194.2007 18042832

[19] Neschen S Katterle Y Richter J Augustin R Scherneck S Mirhashemi F. Uncoupling protein 1 expression in murine skeletal muscle increases AMPK activation, glucose turnover, and insulin sensitivity in vivo. Physiol Genomics (2008) 33:333–40. doi: 10.1152/physiolgenomics.00226.2007 18349383

[20] Keipert S Ost M Chadt A Voigt A Ayala V Portero-Otin M. Skeletal muscle uncoupling-induced longevity in mice is linked to increased substrate metabolism and induction of the endogenous antioxidant defense system. Am J Physiol Endocrinol Metab (2013) 304:E495–506. doi: 10.1152/ajpendo.00518.2012 23277187

[21] Masania J Wijten P Keipert S Ost M Klaus S Rabbani N. Decreased methylglyoxal-mediated protein glycation in the healthy aging mouse model of ectopic expression of UCP1 in skeletal muscle. Redox Biol (2023) 59:102574. doi: 10.1016/j.redox.2022.102574 36521306 PMC9772855

[22] Ost M Coleman V Voigt A van Schothorst EM Keipert S van der Stelt I. Muscle mitochondrial stress adaptation operates independently of endogenous FGF21 action. Mol Metab (2016) 5:79–90. doi: 10.1016/j.molmet.2015.11.002 26909316 PMC4735627

[23] Ost M Igual Gil C Coleman V Keipert S Efstathiou S Vidic V. Muscle-derived GDF15 drives diurnal anorexia and systemic metabolic remodeling during mitochondrial stress. EMBO Rep (2020) 21(3):e48804. doi: 10.15252/embr.201948804 32026535 PMC7054681

[24] Igual Gil C Coull BM Jonas W Lippert RN Klaus S Ost M. Mitochondrial stress-induced GFRAL signaling controls diurnal food intake and anxiety-like behavior. Life Sci Alliance (2022) 5(11):e202201495. doi: 10.26508/lsa.202201495 36271504 PMC9449705

[25] Gil CI Coull BM Jonas W Lippert R Ost M Klaus S. Mitochondrial stress-induced GDF15-GFRAL axis promotes anxiety-like behavior and CRH-dependent anorexia. bioRxiv (2021). doi: 10.1101/2021.09.22.461199

[26] Florian S Krehl S Loewinger M Kipp A Banning A Esworthy S. Loss of GPx2 increases apoptosis, mitosis, and GPx1 expression in the intestine of mice. Free Radic Biol Med (2010) 49:1694–702. doi: 10.1016/j.freeradbiomed.2010.08.029 PMC4132893 20828612

[27] Müller M Banning A Brigelius-Flohé R Kipp A. Nrf2 target genes are induced under marginal selenium-deficiency. Genes Nutr (2010) 5:297–307. doi: 10.1007/s12263-010-0168-8 21189866 PMC2989369

[28] Lossow K Schlörmann W Tuchtenhagen M Schwarz M Schwerdtle T Kipp AP. Measurement of trace elements in murine liver tissue samples: Comparison between ICP-MS/MS and TXRF. J Trace Elements Med Biol (2023) 78:127167. doi: 10.1016/j.jtemb.2023.127167 37004477

[29] Weber D Stuetz W Bernhard W Franz A Raith M Grune T. Oxidative stress markers and micronutrients in maternal and cord blood in relation to neonatal outcome. Eur J Clin Nutr (2014) 68:215–22. doi: 10.1038/ejcn.2013.263 24327121

[30] Dyar KA Ciciliot S Wright LE Bienso RS Tagliazucchi GM Patel VR. Muscle insulin sensitivity and glucose metabolism are controlled by the intrinsic muscle clock. Mol Metab (2014) 3:29–41. doi: 10.1016/j.molmet.2013.10.005 24567902 PMC3929910

[31] Dyar KA Hubert MJ Mir AA Ciciliot S Lutter D Greulich F. Transcriptional programming of lipid and amino acid metabolism by the skeletal muscle circadian clock. PloS Biol (2018) 16:e2005886. doi: 10.1371/journal.pbio.2005886 30096135 PMC6105032

[32] Keipert S Ost M. Stress-induced FGF21 and GDF15 in obesity and obesity resistance. Trends Endocrinol Metab (2021) 32(11):904–15. doi: 10.1016/j.tem.2021.08.008 34526227

[33] Xie Y Kang R Klionsky DJ Tang D. GPX4 in cell death, autophagy, and disease. Autophagy (2023) 19(10):2621–38. doi: 10.1080/15548627.2023.2218764 PMC10472888 37272058

[34] Coleman V Sa-Nguanmoo P Koenig J Schulz TJ Grune T Klaus S. Partial involvement of Nrf2 in skeletal muscle mitohormesis as an adaptive response to mitochondrial uncoupling. Sci Rep (2018) 8:2446. doi: 10.1038/s41598-018-20901-4 29402993 PMC5799251

[35] Ingold I Berndt C Schmitt S Doll S Poschmann G Buday K. Selenium utilization by GPX4 is required to prevent hydroperoxide-induced ferroptosis. Cell (2018) 172:409–422 e21. doi: 10.1016/j.cell.2017.11.048 29290465

[36] Schwarz M Loser A Cheng Q Wichmann-Costaganna M SChadel P Werz O. Side-by-side comparison of recombinant human glutathione peroxidases identifies overlapping substrate specificities for soluble hydroperoxides. Redox Biol (2023) 59:102593. doi: 10.1016/j.redox.2022.102593 36608588 PMC9827380

[37] Wang Y Zhang Z Jiao W Wang Y Wang X Zhao Y. Ferroptosis and its role in skeletal muscle diseases. Front Mol Biosci (2022) 9:1051866. doi: 10.3389/fmolb.2022.1051866 36406272 PMC9669482

[38] Tang D Chen X Kang R Kroemer G. Ferroptosis: molecular mechanisms and health implications. Cell Res (2021) 31:107–25. doi: 10.1038/s41422-020-00441-1 PMC8026611 33268902

[39] Theil EC. Ferritin: the protein nanocage and iron biomineral in health and in disease. Inorg Chem (2013) 52:12223–33. doi: 10.1021/ic400484n PMC3882016 24102308

[40] Keipert S Klaus S Heldmaier G Jastroch M. UCP1 ectopically expressed in murine muscle displays native function and mitigates mitochondrial superoxide production. Biochim Biophys Acta (2010) 1797:324–30. doi: 10.1016/j.bbabio.2009.11.008 19958747

[41] Hirschenson J Melgar-Bermudez E Mailloux RJ. The uncoupling proteins: A systematic review on the mechanism used in the prevention of oxidative stress. Antioxidants (Basel) (2022) 11(2):322. doi: 10.3390/antiox11020322 35204205 PMC8868465

[42] Klaus S Igual Gil C Ost M. Regulation of diurnal energy balance by mitokines. Cell Mol Life Sci (2021) 78:3369–84. doi: 10.1007/s00018-020-03748-9 PMC7814174 33464381

[43] Sato T Sato S. Circadian regulation of metabolism - commitment to health and diseases. Endocrinology (2023) 164(7):bqad086. doi: 10.1210/endocr/bqad086 37253106

[44] Mahboobifard F Pourgholami MH Jorjani M Dargahi L Amiri M Sadeghi S. Estrogen as a key regulator of energy homeostasis and metabolic health. BioMed Pharmacother (2022) 156:113808. doi: 10.1016/j.biopha.2022.113808 36252357

[45] Riley LG Nafisinia M Menezes MJ Nambiar R Williams A Barnes EH. FGF21 outperforms GDF15 as a diagnostic biomarker of mitochondrial disease in children. Mol Genet Metab (2022) 135:63–71. doi: 10.1016/j.ymgme.2021.12.001 34991945

[46] Maresca A Del Dotto V Romagnoli M La Morgia C Di Vito L Capristo M. Expanding and validating the biomarkers for mitochondrial diseases. J Mol Med (Berl) (2020) 98(10):1467–78. doi: 10.1007/s00109-020-01967-y PMC7524861 32851462

[47] Lehtonen JM Forsstrom S Bottani E Viscomi C Baris OR Isoniemi H. FGF21 is a biomarker for mitochondrial translation and mtDNA maintenance disorders. Neurology (2016) 87:2290–9. doi: 10.1212/WNL.0000000000003374 PMC5270510 27794108

[48] Conte M Sabbatinelli J Chiariello A Martucci M Santoro A Monti D. Disease-specific plasma levels of mitokines FGF21, GDF15, and Humanin in type II diabetes and Alzheimer’s disease in comparison with healthy aging. GeroScience (2021) 43:985–1001. doi: 10.1007/s11357-020-00287-w 33131010 PMC8110619

[49] Ahola S Rivera Mejías P Hermans S Chandragiri S Giavalisco P Nolte H. OMA1-mediated integrated stress response protects against ferroptosis in mitochondrial cardiomyopathy. Cell Metab (2022) 34:1875–1891.e7. doi: 10.1016/j.cmet.2022.08.017 36113464

[50] Ahola S Langer T. Ferroptosis in mitochondrial cardiomyopathy. Trends Cell Biol (2023) S0962-8924(23)00125-3. doi: 10.1016/j.tcb.2023.06.002 37419738

[51] Gardner PR. Aconitase: sensitive target and measure of superoxide. Methods Enzymol (2002) 349:9–23. doi: 10.1016/S0076-6879(02)49317-2 11912933

[52] Gao M Yi J Zhu J Minikes AM Monian P Thompson CB. Role of mitochondria in ferroptosis. Mol Cell (2019) 73:354–363.e3. doi: 10.1016/j.molcel.2018.10.042 30581146 PMC6338496

[53] Tian-Xiang W Kun-Long D Zi-Xuan H Zi-An X Jun-Yun L Yongjun D. Tanshinone functions as a coenzyme that confers gain of function of NQO1 to suppress ferroptosis. Life Sci Alliance (2023) 6:e202201667. doi: 10.26508/lsa.202201667 36319062 PMC9629850

[54] Anahita O Stefan K Imke LL Daniel TH Nienke W Batoul B. Activating the NFE2L1-ubiquitin-proteasome system by DDI2 protects from ferroptosis. bioRxiv (2023). doi: 10.1101/2023.07.04.547652

[55] Couplan E Gelly C Goubern M Fleury C Quesson B Silberberg M. High level of uncoupling protein 1 expression in muscle of transgenic mice selectively affects muscles at rest and decreases their IIb fiber content. J Biol Chem (2002) 277:43079–88. doi: 10.1074/jbc.M206726200 12221093

[56] Kreß JKC Jessen C Hufnagel A Schmitz W Xavier da Silva TN Ferreira dos Santos A. The integrated stress response effector ATF4 is an obligatory metabolic activator of NRF2. Cell Rep (2023) 42:112724. doi: 10.1016/j.celrep.2023.112724 37410595

[57] Stockwell BR. Ferroptosis turns 10: Emerging mechanisms, physiological functions, and therapeutic applications. Cell (2022) 185:2401–21. doi: 10.1016/j.cell.2022.06.003 PMC9273022 35803244

[58] Yang WS Kim KJ Gaschler MM Patel M Shchepinov MS Stockwell BR. Peroxidation of polyunsaturated fatty acids by lipoxygenases drives ferroptosis. Proc Natl Acad Sci U.S.A. (2016) 113:E4966–75. doi: 10.1073/pnas.1603244113 PMC5003261 27506793

[59] Conrad M Sato H. The oxidative stress-inducible cystine/glutamate antiporter, system x (c) (-) : cystine supplier and beyond. Amino Acids (2012) 42:231–46. doi: 10.1007/s00726-011-0867-5 21409388

[60] Doll S Proneth B Tyurina YY Panzilius E Kobayashi S Ingold I. ACSL4 dictates ferroptosis sensitivity by shaping cellular lipid composition. Nat Chem Biol (2017) 13:91–8. doi: 10.1038/nchembio.2239 PMC5610546 27842070

[61] Zou Y Henry WS Ricq EL Graham ET Phadnis VV Maretich P. Plasticity of ether lipids promotes ferroptosis susceptibility and evasion. Nature (2020) 585:603–8. doi: 10.1038/s41586-020-2732-8 PMC8051864 32939090

