# Supplementary figures and images for: Temporal dynamics of muscle mitochondrial uncoupling-induced integrated stress response and ferroptosis defense
Source: Front Endocrinol (Lausanne). 2023 Oct 23;14:1277866. doi: 10.3389/fendo.2023.1277866 (PMC10627798; doi:10.3389/fendo.2023.1277866)

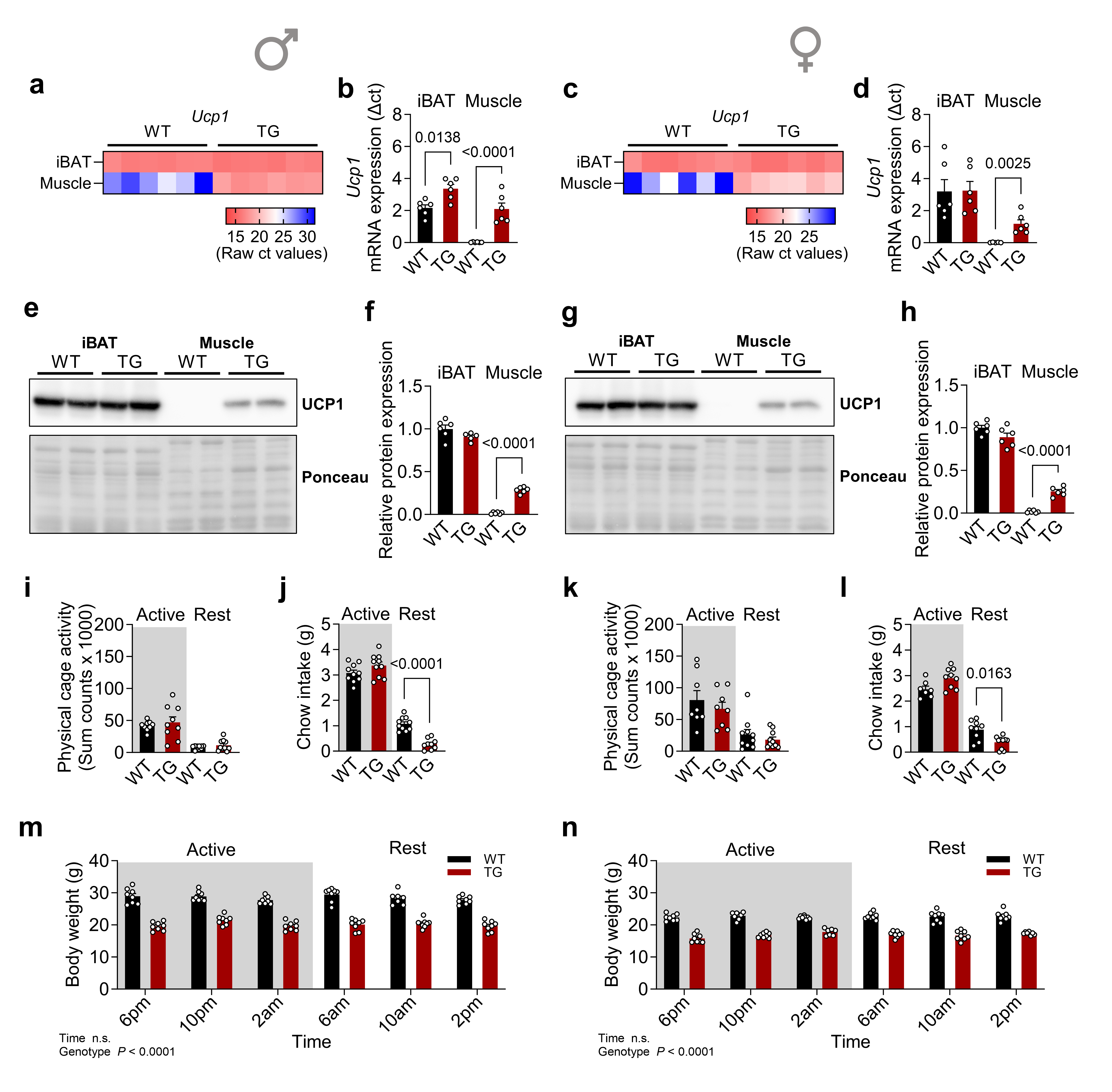

Supplement: Supplementary Figure 1 — Phenotypic characterization of mUcp1-transgenic mice. (A-D) Ucp1 mRNA expression in interscapular BAT (iBAT) and skeletal muscle represented as raw ct values (A, C) and Δct values using B2m as housekeeping gene (B, D) in male and female mice, respectively (n=6 per genotype, sex and tissue). (E, G) Representative immunoblots and (F, H) quantification of UCP1 expression in iBAT and muscle in male and female mice, respectively. Relative protein expression was calculated normalizing raw intensity values to iBAT WT samples (n= 5-6 per genotype, sex and tissue). (I-L) Quantification of total active- versus inactive phase physical activity (I, K) and chow intake (J, L) in male and female mice, respectively (n=7-10 per genotype and sex). (M, N) Body weight of mice sacrificed during a 24-hour period in 4-hour intervals corresponding to (n=7-8 per genotype and sex). WT, wildtype; TG, mUcp1-transgenic. Data were analyzed using a one-way ANOVA (B, D, F, H, I-L) or two-way ANOVA with the Geisser-Greenhouse correction (m, n). Unless otherwise indicated, data correspond to mice sacrificed at 10 am. [file Image_1.tif]

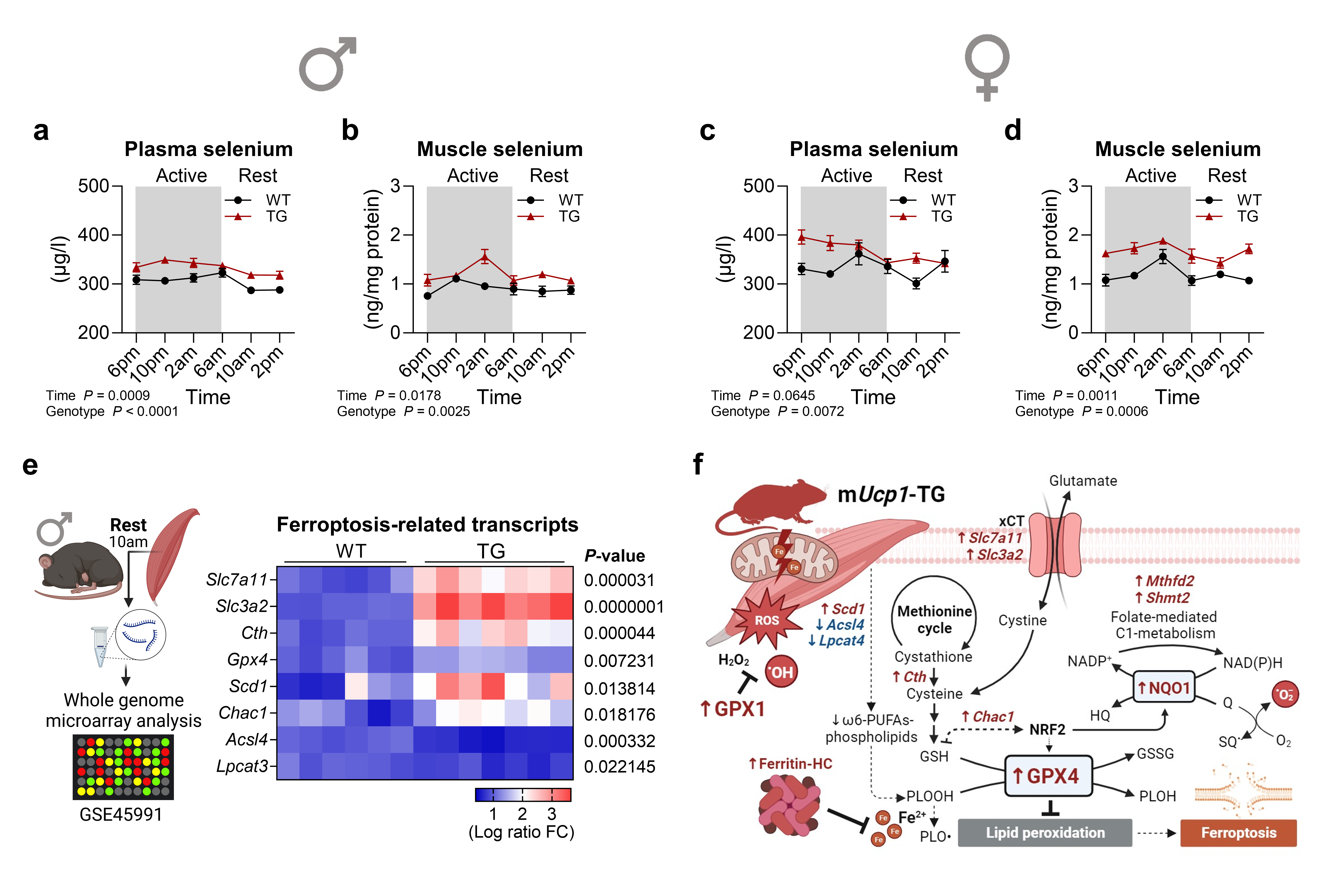

Supplement: Supplementary Figure 2 — Temporal variation of plasma and muscle selenium. (A, C) Plasma (n=7-8 per genotype, sex and timepoint) and (B, D) skeletal muscle (n=4-5 per genotype, sex and timepoint) selenium concentration in male and female mice, respectively. Samples were harvested during a 24-hour period in 4-hour intervals. WT, wildtype; TG, mUcp1-transgenic. Data were analyzed using a two-way ANOVA with the Geisser-Greenhouse correction. (E) Heat map of ferroptosis related genes extracted from a published microarray analysis (GSE45991) of skeletal muscle in male TG mice collected at 10am. (F) Suggested mechanism of oxidative defense and ferroptosis signature in skeletal muscle of TG mice. Genes/proteins shown in red are upregulated while those shown in blue are downregulated in TG muscle compared to WT. [file Image_2.tif]
